# Genetic Susceptibility to *Helicobacter pylori* Infection and Pancreatic Cancer Risk: A Two-Sample Mendelian Randomisation Study

**DOI:** 10.3390/life16020284

**Published:** 2026-02-07

**Authors:** Nien-Yu Yang, Te-Min Ke, Yicong Huang, Artitaya Lophatananon, Kenneth R. Muir

**Affiliations:** 1 Division of Population Health, Health Services Research and Primary Care, School of Health Sciences, Faculty of Biology, Medicine and Health, The University of Manchester, Manchester M13 9PT, UK; sunny.yang@manchester.ac.uk (N.-Y.Y.); yicong.huang@manchester.ac.uk (Y.H.); artitaya.lophatananon@manchester.ac.uk (A.L.); 2 Department of Radiation Oncology, Chi Mei Medical Center, No. 901, Zhonghua Road, Yongkang District, Tainan City 710, Taiwan; b30932@mail.chimei.org.tw

**Keywords:** pancreatic cancer, *Helicobacter pylori*, two-sample Mendelian randomisation, causal inference, genetic epidemiology

## Abstract

**Background/Objectives**: Pancreatic cancer is one of the most lethal malignancies, with poor survival and few established modifiable risk factors. While *Helicobacter pylori* (*H. pylori*) infection is a known cause of gastric cancer, its role in pancreatic cancer remains unclear, with inconsistent observational evidence. **Methods**: We applied two-sample Mendelian randomisation (2SMR) to assess the causal effect of *H. pylori* infection on pancreatic cancer risk. Genetic instruments were derived from GWAS data on anti-*H. pylori* IgG levels in the ALSPAC cohort (n = 4638). Outcomes were pancreatic cancer cases from UK Biobank (936 cases, 400,294 controls) and a combined dataset including UK Biobank, FinnGen, and MVP (5979 cases, 1,234,860 controls). Inverse-variance weighted (IVW) MR was the primary method, supported by MR-Egger, weighted median/mode and MR-PRESSO, with sensitivity analyses for pleiotropy. **Results**: No significant causal association was observed. IVW ORs were 1.039 (95% CI: 0.846–1.440, *p* = 0.466) for UK Biobank and 1.077 (95% CI: 0.962–1.206, *p* = 0.197) for the combined dataset. All complementary methods yielded null results, with no strong evidence of pleiotropy. **Conclusions**: This 2SMR study found no evidence that *H. pylori* infection causally increases pancreatic cancer risk. Larger studies with refined exposure measures are warranted.

## 1. Introduction

Pancreatic cancer (PaCa) represents one of the most formidable malignancies, characterised by rapidly increasing incidence rates and persistently poor survival outcomes. Globally, pancreatic cancer ranks as the twelfth most common cancer and the seventh leading cause of cancer-related mortality, with statistics predicting substantial increases in both incidence (78.3%) and mortality (81.9%) from 2022 to 2045 [1,2,3]. In the United Kingdom, pancreatic cancer is the tenth most prevalent cancer, accounting for approximately 3% of all cancer cases with around 10,452 annual diagnoses. It also ranks as the fifth leading cause of cancer deaths, representing 6% of total cancer mortality [4]. The five-year survival rate remains devastatingly low at approximately 7%, largely due to late-stage diagnosis in the absence of specific symptoms or effective screening programs [5].

Previous research has successfully developed an integrative pancreatic cancer risk prediction model using UK Biobank data, demonstrating the value of combining lifestyle, genetic, and medical history variables for accurate risk assessment [6]. To identify further factors and explore their causal relationship with PaCa, this present study applies two-sample Mendelian randomisation (2SMR) to evaluate the potential causal role of *Helicobacter pylori* (*H. pylori*) infection.

*H. pylori* was chosen for this investigation due to its established role as a risk factor for stomach cancer and the limited, though inconsistent, evidence suggesting a possible association with increased pancreatic cancer risk. This indicates *H. pylori*’s potential as a shared risk factor across different cancer types, particularly stomach and pancreatic cancer, requiring further investigation [7]. Additionally, family history of stomach cancer has been associated with modestly increased pancreatic cancer mortality, and this link could potentially be mediated by shared *Helicobacter pylori* infections within families [8,9]. The integrative pancreatic cancer risk prediction model in the UK Biobank, while not finding a significant difference in the distribution of *H. pylori* infection between cases and controls, nonetheless included it as a medical history-related variable for consideration [6]. By identifying modifiable factors with established causal effects on pancreatic cancer development, we aim to uncover new leads for PaCa prevention, further supporting more targeted prevention strategies and advancing population-level cancer prognosis.

MR is an epidemiological methodology that introduces genetic variants as instrumental variables (IVs) to investigate causal associations between risk factors and health outcomes [10,11,12]. This approach offers distinct advantages over conventional observational research because genetic variants are randomly distributed during conception [10,11,12], similar to the randomisation process in the experimental studies. However, the validity of MR builds on three fundamental criteria [13]. To begin with, the IVs must demonstrate a strong association with the exposure of interest (relevance criterion). Second of all, the genetic instruments must not be associated with any confounders of the relationship between the instrument and the outcome (independence criterion), and lastly, the genetic instruments should affect the outcome exclusively through their influence on the exposure variable (exclusion restriction criterion) [13]. With the assumptions being fulfilled, MR can minimise confounding and mitigate reverse causation, allowing for causal inference comparable to that of a randomised controlled trial [10,11,12].

Two-sample MR offers a methodological advancement within this framework. In contrast to conventional MR approaches that typically require individual-level genetic and phenotypic data from identical study populations, the two-sample method can utilise summary statistics derived from independent genome-wide association studies conducted on different cohorts for exposure and outcome variables, respectively [14,15]. This approach significantly enhances the feasibility and scope of MR analyses by allowing researchers to investigate causal relationships.

To investigate a potential new lead in pancreatic cancer (PaCa) and support future public health prevention strategies, it is important to assess the possible causal role of *H. pylori* infection. Although some observational studies have suggested an association between *H. pylori* and PaCa, the evidence remains limited and inconsistent. Given its established role in gastric cancer and potential relevance to PaCa, a more robust causal analysis is needed [7,8,9]. In this study, we conducted a two-sample Mendelian randomisation (2SMR) analysis to determine whether genetic susceptibility to *H. pylori* infection is causally linked to pancreatic cancer risk. Genome-wide association study (GWAS) summary statistics from large, population-based datasets, including FinnGen, MVP (EUR), and UK Biobank, were utilised to examine this potential relationship.

## 2. Materials and Methods

### 2.1. Study Design

The study applied a two-sample Mendelian randomisation (2SMR) approach. Genetic instruments for the exposure were selected from publicly available genome-wide association study (GWAS) summary statistics related to anti-*H. pylori* IgG levels, sourced from the IEU Open GWAS Project (https://gwas.mrcieu.ac.uk/datasets/ieu-b-4905/, accessed on 12 March 2025). For the pancreatic cancer outcome, GWAS summary data were obtained from three large population-based cohorts: FinnGen, MVP (EUR), and the UK Biobank. All summary statistics related to SNP outcome were obtained from https://mvp-ukbb.finngen.fi/. Details of the number of pancreatic cancer outcomes for each dataset are available at https://mvp-ukbb.finngen.fi/. The study was conducted in accordance with the three core assumptions of Mendelian randomisation [16]: (1) the genetic variants used as instruments must be strongly associated with *H. pylori* exposure; (2) they must not be associated with variables that confound the relationship between the genetic instruments and the outcome; and (3) they must influence pancreatic cancer risk solely through their effect on *H. pylori* infection.

### 2.2. Genetic Instrument Selection

**Figure 1:**
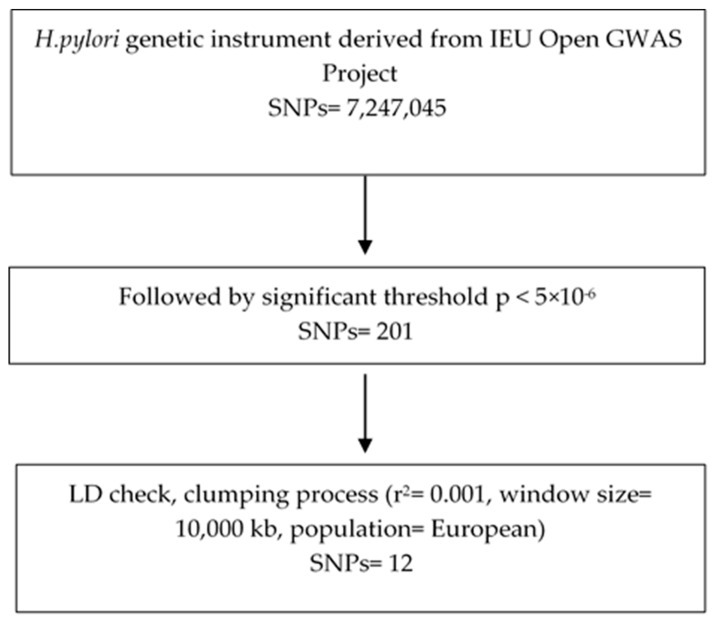
The flowchart for *H. pylori* instrument selection. Genetic instrument sourced from IEU Open GWAS Project.

Genetic instruments for *Helicobacter pylori* infection were selected from the IEU Open GWAS Project, using summary statistics from dataset ieu-b-4905, which was derived from the ALSPAC Clinic—Focus@7 cohort [17]. This GWAS, based on anti-*H. pylori* IgG levels, included 4638 individuals and 7,247,045 single-nucleotide polymorphisms (SNPs). Variants with genome-wide significance at *p* < 5 × 10^−6^ were initially considered as candidate instrumental variables (n = 201). To ensure independence among SNPs, linkage disequilibrium (LD) clumping was applied using an r^2^ threshold of 0.001 within a 10,000 kb window, based on the 1000 Genomes Project Phase 3 European reference panel (HG19/GRCh37) [17]. This resulted in the identification of 12 independent SNPs. Figure 1 depicts the instrument selection process.

### 2.3. Outcome Data Source

Pancreatic cancer GWAS summary data were obtained from the R12 release of the FinnGen Consortium, which combined FinnGen, MVP (EUR) and UK Biobank data. We selected outcomes using “Malignant neoplasm of pancreas, excluding all cancers (controls excluding all cancers)”. Details are available at: https://storage.googleapis.com/finngen-public-data-r12/meta_analysis/mvp_ukbb/summary_stats_filtered/C3_PANCREAS_EXALLC_meta_out_filtered.tsv.gz (accessed 20 March 2025). This database comprises 3139 cases and 378,749 controls from FinnGen, 1904 cases and 455,817 controls from MVP(EUR), and 936 cases and 400,294 controls from the UK Biobank. Table S6 provides detailed descriptions of the pancreatic cancer GWAS dataset characteristics.

### 2.4. The Strength of the Selection of Instrumental Variables and Power Calculations

To mitigate weak instrument bias, instrument strength was assessed by two metrics. The proportion of variance explained (R^2^) was introduced to evaluate overall strength, while the F-statistic was adopted to measure individual instrument strength [18]. R^2^ refers to how much exposure variance can be accounted for by the selected genetic variants, with higher values improving Mendelian randomisation effectiveness [18]. The F-statistic evaluates instrumental variable robustness, where values under 10 suggest inadequate instrument strength [19,20]. *PVE* refers to the proportion of variance in the exposure explained by the genetic instrument(s) (also commonly denoted as R^2^) [21]. For a single biallelic genetic variant, *PVE* is calculated as:

*PVE* = 2 × *EAF* × (1 − *EAF*) × *β*^2^

where *EAF* is the effect allele frequency and *β* is the estimated per-allele effect of the genetic variant on the exposure, expressed on the appropriate scale. When multiple genetic variants are used as instruments, the total *PVE* is calculated as the sum of the *PVE* values across all variants, assuming independence between instruments.

The *F*-statistic is then derived from the PVE and reflects overall instrument strength:

*F* = *PVE* × (*N* − *K* − 1)/1 − *PVE*) × *K*

where *N* is the sample size and *K* is the number of genetic instruments. Consistent with established guidance, F-statistics below 10 indicate weak instruments and potential bias in Mendelian randomisation analyses.

### 2.5. Two-Sample Mendelian Randomisation (2SMR) Analysis

Multiple complementary approaches were used to perform two-sample Mendelian randomisation analysis across two different outcome datasets to enhance statistical power. The inverse-variance weighted (IVW) method [22] served as our primary analytical approach, supplemented by five robust techniques: MR-Egger [23], weighted median (WM) [24], weighted mode (WMO) [25] and MR-PRESSO [26].

The IVW method combines individual SNP effect estimates to generate an overall causal estimate [22]. This approach requires valid instrumental variables that meet all three Mendelian randomisation assumptions [22]. Given its statistical efficiency with appropriate instruments, IVW is typically the preferred primary method. However, additional MR methods are necessary to identify potential pleiotropy [27].

MR-Egger estimates causal effects by including the average pleiotropy as the intercept [27]. Its reliability depends on the assumption that the genetic variants’ indirect effects are not related to the exposure, known as the Instrument Strength Independent of Direct Effect (InSIDE) assumption [23]. The WM approach is based on a majority valid assumption, which considers that most variants are valid instruments [24]. The WM method weighs each variant’s outcome effect by its exposure association strength before determining the median. This method effectively deals with invalid instruments and outliers [27].

The WMO method builds on the plurality valid assumption, which assumes that the largest weighted cluster of variants reflects the true causal effect [27]. Like the weighted median approach, it assigns weights to each SNP; however, it uses the mode, rather than the median, as the central estimate. Both mode-based methods are considered robust against the influence of invalid instruments and outliers [25,28].

MR-PRESSO extends IVW by incorporating global, outlier, and distortion tests [26]. The global test detects horizontal pleiotropy [27], and it removes outliers when horizontal pleiotropy is detected. In addition, the distortion test evaluates whether effect estimates differ significantly before and after outlier correction [26].

Our analytical strategy utilised IVW as the primary method, supplemented by five complementary approaches. Initially, 12 selected *H. pylori* SNPs were analysed against UKBB pancreatic cancer outcome data. To enhance statistical power, a second analysis examined 11 *H. pylori* instruments against the combined FinnGen, MVP (EUR), and UKBB datasets, excluding rs117912702 due to its absence from MVP. This combined approach utilised larger sample sizes and diverse population characteristics to strengthen our causal inferences.

Identical sensitivity assessments were applied to both analyses, including MR-Egger intercept tests for horizontal pleiotropy (*p* < 0.05), funnel plot visualisation for asymmetric patterns [23], and Cochran’s Q test for heterogeneity (*p* < 0.05). MR Steiger directionality testing [29] validated causal direction by confirming that genetic instruments explained less variance in outcomes than exposures. Finally, MR-PRESSO was performed to detect outliers and horizontal pleiotropy.

**Figure 2:**
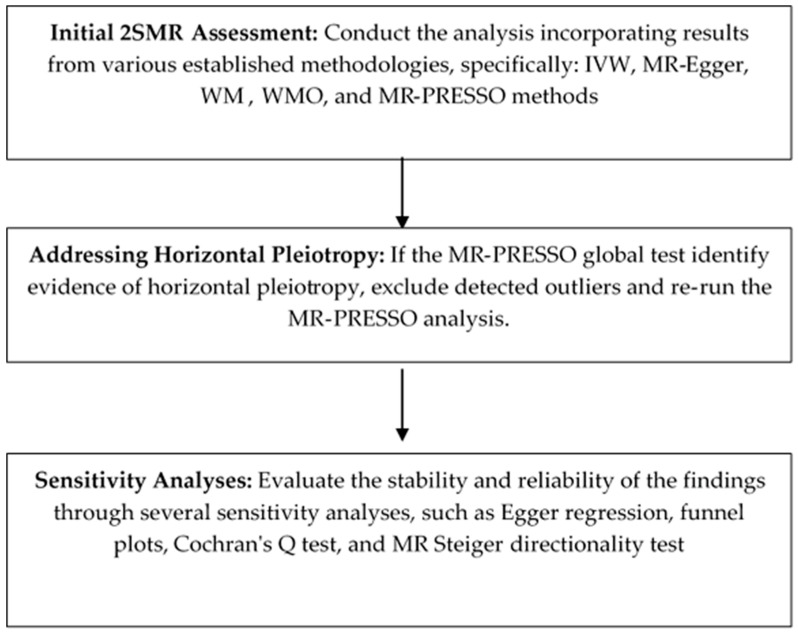
Procedure for two-sample Mendelian randomisation (2SMR) analysis.

The analysis adhered to STROBE-MR reporting guidelines [30]. The complete analytical workflow is illustrated in Figure 2.

### 2.6. Statistical Analysis

Statistical significance was defined as *p* < 0.05, with 95% confidence intervals not encompassing one. All analyses were performed using R software [31] (version 4.4.3, R Development Core Team, Vienna, Austria). We employed the TwoSampleMR version 0.6.29 [14] and MR-PRESSO version 1.0 [32] R packages for two-sample MR analyses, utilising functions including harmonise_data, mr, mr_presso, mr_heterogeneity, mr_pleiotropy_test, mr_singlesnp, mr_scatter_plot, mr_forest_plot, mr_funnel_plot, and directionality_test [33].

### 2.7. Ethics

This study utilised publicly available GWAS summary statistics exclusively. Since no new data were collected, no additional ethical approval was required.

## 3. Results

### 3.1. Selection of Instrumental Variables

Following the instrumental variable selection protocol described in Section 2.2, 12 independent SNPs were identified. These SNPs were obtained from the IEU Open GWAS Project (dataset ieu-b-4905), which was derived from the ALSPAC Clinic—Focus@7 cohort [17], and were used in the comprehensive Mendelian randomisation analysis. Full details of the selected instrumental variables are provided in Supplementary Table S1. The total R^2^ was 0.06% and the mean F-statistic across these 12 instruments was 22.37 (range: 21.08–26.10). Due to the absence of rs117912702 in the MVP dataset, 11 instrumental variables were used in the combined analysis, leading to a total R^2^ of 0.05% and a mean F-statistic of 22.42 (range: 21.08–26.10). Full details of the selected instrumental variables for both analyses are provided in Supplementary Tables S1 and S2. We also investigated the FinnGen and MVP Mendelian randomisation analyses separately. The instrumental variables used for the FinnGen and MVP analyses are summarised in Tables S3 and S4, and the corresponding Mendelian randomisation results are provided in Table S5.

### 3.2. MR Analysis

Results from the two-sample Mendelian randomisation analyses are shown in Table 1. Analysis of the UK Biobank dataset revealed no significant causal link between *H. pylori* infection and pancreatic cancer risk across all Mendelian randomisation methods. The primary inverse-variance weighted (IVW) method showed an odds ratio (OR) of 1.039 (95% CI = 0.846–1.440, *p* = 0.466). The weighted mode (OR = 1.222, 95% CI = 0.688–2.117, *p* = 0.508) also indicated non-significant associations. The MR-Egger method resulted in an OR of 0.942 (95% CI = 0.464–1.914, *p* = 0.872), while the weighted median approach further supported the absence of a significant causal association with an OR of 1.172 (95% CI = 0.834–1.647, *p* = 0.361). MR-PRESSO analysis did not detect a significant causal effect (OR = 1.104, 95% CI: 0.846–1.440, *p* = 0.481).

In a combined dataset including FinnGen, UK Biobank, and MVP participants of European ancestry, with 11 instrumental variables due to the absence of rs117912702 in the MVP dataset, all Mendelian randomisation models consistently showed no significant causal relationship between *H. pylori* exposure and pancreatic cancer risk, as none of the *p*-values were below 0.05. The IVW method resulted in an OR of 1.077 (95% CI: 0.962–1.206, *p* = 0.197) and the MR-Egger regression returned an OR of 0.990 (95% CI: 0.727–1.347, *p* = 0.950). Similarly, the weighted median result was non-significant (OR = 1.073, 95% CI: 0.938–1.227, *p* = 0.308). Estimates from the weighted mode (OR = 1.147, 95% CI: 0.893–1.474, *p* = 0.304) were consistent with these findings. Furthermore, MR-PRESSO did not detect pleiotropic outliers, and the raw MR estimate was non-significant (*p* = 0.226).

**Figure 3:**
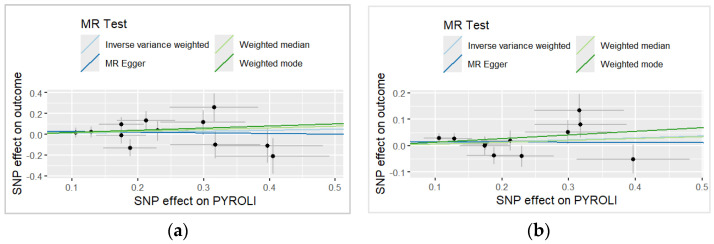
Two-sample MR scatter plots of the causal relationships between *H. pylori* and pancreatic cancer using four MR methods: inverse-variance weighted (light blue), MR Egger (blue), weighted median (light green) and weighted mode (green). (**a**) Results based on the UK Biobank (UKBB) dataset. (**b**) Results based on the combined dataset. Each point represents an individual SNP with corresponding standard errors. Regression lines indicate the estimated causal effect under each MR method.

In the scatter plots (Figure 3), the direction of the causal effect of *H. pylori* on the outcome was consistent across all MR analysis methods in both the UKBB and the combined dataset. The MR estimates in the combined dataset showed a more distinct positive slope compared to the UKBB-only analysis.

**Table 1:** Summary of two-sample MR analyses.

PaCa Outcome Source	MR Method	nsnp	OR	*p*-Value	95% CI
**UKBB**	WMO	12	1.222	0.508	0.688–2.171
	WM	12	1.172	0.361	0.834–1.647
	MR Egger	12	0.942	0.872	0.464–1.914
	IVW	12	1.039	0.466	0.846–1.440
	MR-PRESSO	12	1.104	0.481	0.846–1.440
**FinnGen + MVP (EUR) + UKBB**	WMO	11	1.147	0.308	0.893–1.474
	WM	11	1.073	0.305	0.938–1.227
	MR Egger	11	0.990	0.950	0.727–1.347
	IVW	11	1.077	0.197	0.962–1.206
	MR-PRESSO	11	1.077	0.226	0.962–1.206

PaCa: pancreatic cancer; MR: Mendelian randomisation; nsnp: number of single-nucleotide polymorphisms; OR: odds ratio; 95% CI: 95% confidence interval; UKBB: UK Biobank; MR-Egger: Mendelian randomisation–Egger method; WM: weighted median; IVW: inverse-variance weighted; WMO: weighted mode; MR-PRESSO: Mendelian Randomisation Pleiotropy RESidual Sum and Outlier.

### 3.3. Sensitivity Analysis

**Figure 4:**
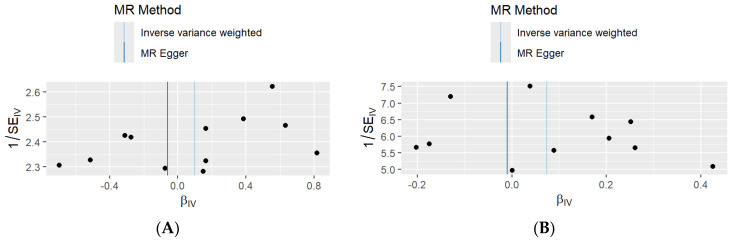
Funnel plots from two-sample Mendelian randomisation analyses assessing the causal effect of *H. pylori* infection on pancreatic cancer risk. (**A**) Results based on the UKBB outcome dataset. (**B**) Results based on the FinnGen, MVP(EUR), and UKBB outcome datasets.

The intercept analysis using MR-Egger regression demonstrated an absence of directional horizontal pleiotropy in the UKBB outcome database, as the intercept (0.033, SE = 0.070) did not significantly differ from zero (*p* = 0.644). However, the funnel plot demonstrated in Figure 4A,B. Both figures showed some asymmetrical patterns in the distribution of SNP-specific causal estimates around the IVW estimate. The instrumental variables are distributed across a range of βIV values from approximately –0.4 to 0.8, with corresponding 1/SEIV values clustering between 2.3 and 2.6. Despite this asymmetry, the MR-Egger and IVW estimates were closely aligned.

When analysing the combined outcome database, the MR-Egger intercept was estimated at 0.017 (SE = 0.030, *p* = 0.577), showing no significant deviation from zero. The funnel plot (Figure 4B) displayed a more symmetrical distribution of SNP-specific βIV values, ranging from approximately −0.2 to 0.4, with inverse standard error values spanning from 5.0 to 7.5. The estimates from the inverse-variance weighted and MR-Egger methods were closely aligned.

Cochran’s Q test for heterogeneity showed a *p*-value of >0.05, suggesting no heterogeneity from different genetic instruments.

## 4. Discussion

To explore the causal relationship between *H. pylori* infection and pancreatic cancer risk, we performed a two-sample Mendelian randomisation analysis using independent genetic instruments from the IEU Open GWAS Project and outcome data from the UK Biobank and a combined dataset comprising FinnGen, MVP (EUR), and UKBB. The inverse-variance weighted method, along with five other robust methods, was utilised in the MR analysis. Sensitivity analyses, including MR-Egger intercept tests, funnel plots, and MR-PRESSO, were conducted to strengthen our results. Our findings revealed that genetic liability to *H. pylori* infection was not significantly associated with pancreatic cancer risk.

Across both the UK Biobank-only analysis and the combined dataset, including FinnGen, MVP (EUR), and the UKBB, Mendelian randomisation analyses consistently showed no evidence of a causal association between *H. pylori* infection and pancreatic cancer risk. Effect estimates across all MR methods were close to the null, with confidence intervals consistently overlapping unity and no statistically significant results observed. The inclusion of larger, combined datasets improved precision, as reflected by narrower confidence intervals, but did not alter the overall null pattern. None of the *p*-values fell below the 0.05 threshold, reinforcing the lack of a genetic causal association across both individual and pooled cohort analyses.

*H. pylori* has been established as a risk factor for gastric cancer, with some epidemiological evidence supporting this relationship [7,34]. However, its potential role in pancreatic cancer remains less well-characterised. To our knowledge, this is the first Mendelian randomisation study specifically examining the causal relationship between *H. pylori* infection and pancreatic cancer risk. Previous serologic studies have shown mixed results, with some suggesting weak associations [35,36], while others found no increased risk of pancreatic cancer [37].

In two independent two-sample Mendelian randomisation analyses, the genetic instruments explained a small proportion of the variance in *H. pylori* infection (R^2^ = 0.06% in UKBB and 0.05% when combining all three datasets), which is typical for complex infectious phenotypes. Importantly, mean F-statistics exceeded the conventional threshold for weak instruments (mean F = 22.37 and 22.42), indicating adequate instrument strength and reducing the likelihood of weak-instrument bias in our MR estimates [19,20,38]. A key limitation, however, is attributed to the utilisation of SNPs that do not meet the stringent conventional genome-wide significance thresholds (*p* < 5 × 10^−8^). Such instruments increase the risk of misclassification and could potentially cause false-positive associations, especially in smaller GWAS. The inclusion of underpowered SNPs could introduce bias into the MR estimates and compromise our ability to identify true causal relationships. Without strong biological justification for the genetic instruments and thorough testing of key assumptions, these results should be interpreted with caution [39,40].

The small number of pancreatic cancer cases in our dataset presents an additional limitation. Even with strong genetic instruments, this leads to imprecise estimates and wide confidence intervals that may obscure smaller but clinically important effects. This challenge is common in Mendelian randomisation studies with limited sample sizes, where detecting modest effects requires substantially larger datasets to establish reliable associations [41].

Additionally, our exposure data derived from childhood *H. pylori* seropositivity (ALSPAC cohort) may not fully capture adult infection status or the chronic colonisation patterns most relevant to pancreatic cancer [17]. The temporal relationship between childhood exposure and adult cancer development introduces complexity in interpreting causal pathways.

Furthermore, some other biological factors may also explain the lack of a causal association between *H. pylori* infection and pancreatic cancer risk. *H. pylori* is largely confined to the gastric mucosa, where it acts through local inflammatory and epithelial mechanisms that are unlikely to affect the pancreas. Proposed systemic pathways, such as hormonal or inflammatory effects, remain poorly supported and are likely weak. Observational associations with extra-gastric cancers often involve virulent CagA-positive strains; however, our genetic instruments reflect general seropositivity rather than strain-specific exposure, which may mask subtype-specific effects. In addition, the exposure GWAS is based on childhood serology, whereas pancreatic cancer develops in adulthood, and this timing mismatch may further attenuate causal estimates. Finally, any small effect of *H. pylori* may be outweighed by stronger established risk factors, including smoking, diabetes, obesity, and chronic pancreatitis.

Our sensitivity analyses provided several methodological insights. Although the MR-Egger intercept tests indicated no evidence of horizontal pleiotropy in either dataset (*p* = 0.644 for UK Biobank; *p* = 0.577 for the combined analysis), the funnel plot for the UK Biobank analysis displayed asymmetry. This may suggest minor pleiotropy, though the lack of statistical significance implies that the pattern more likely reflects sampling variability, given the relatively small number of pancreatic cancer cases. By contrast, the combined dataset showed a more symmetrical funnel plot, consistent with the greater precision afforded by its larger sample size. Funnel plots, however, should be interpreted with caution in this study because the small number of available instruments limits the ability to visually assess symmetry. With few SNPs, apparent asymmetry can arise from random variation rather than true directional pleiotropy, and therefore, conclusions based on funnel plot inspection are inherently uncertain.

Importantly, the combined analysis also yielded consistently null results across all MR methods, reinforcing the robustness of our main conclusions.

MR-PRESSO analysis detected no pleiotropic outliers, though this may reflect the non-significant nature of our primary findings rather than an absence of pleiotropy [39]. Cochran’s Q test for heterogeneity also suggested no violations of MR assumption (horizontal pleiotropy). This indicates consistency of genetic associations across the meta-analysed outcome datasets.

Although our findings do not support a significant causal relationship between *H. pylori* infection and pancreatic cancer risk, the directional consistency across methods and datasets suggests that this relationship requires continued investigation. The clinical implications remain limited given the non-significant results, but the methodology demonstrates the feasibility of applying 2SMR approaches to pancreatic cancer outcomes.

Future studies should address current limitations through several approaches. Including data from multiple international consortia could substantially increase pancreatic cancer case numbers, enhancing statistical power and precision [41]. Additionally, incorporating more comprehensive *H. pylori* exposure measures, including adult genetic proxies, may better capture relevant biological pathways.

Two-step MR or multivariable MR frameworks using individual-level data could enable more flexible modelling approaches. Integration with other causal inference methods, such as phenome-wide association studies (PheWAS) or mediation analysis, may provide complementary insights into potential mechanistic pathways linking *H. pylori* to pancreatic pathophysiology [41,42,43].

Given the absence of genome-wide significant (*p* < 5 × 10^−8^) genetic instruments for *H. pylori* infection, the findings of this study should not be regarded as definitive and must be interpreted with appropriate caution. This limitation reflects the current state of microbiome and infection-susceptibility GWAS, where sufficiently powered instruments are rarely available, rather than a shortcoming unique to the present analysis. Consequently, the use of a relaxed significance threshold represents a pragmatic necessity within the field. Importantly, as future large-scale GWAS identify robust genome-wide significant variants for *H. pylori*, it will be essential to repeat this Mendelian randomisation analysis using *p* < 5 × 10^−8^ instruments to strengthen causal inference and determine whether the null findings observed here persist with more powerful and biologically specific genetic proxies.

This Mendelian randomisation analysis found no significant causal association between *H. pylori* infection and pancreatic cancer risk. While methodological limitations, particularly limited statistical power due to low outcome sample size, may have constrained our ability to detect modest causal effects, the consistency of null findings across multiple analytical approaches and datasets provides reasonable evidence against a strong causal relationship. Future research with enhanced statistical power and refined exposure measurement may be needed to definitively resolve this question.

To address current limitations, future MR studies could incorporate adult or chronic *H. pylori* infection GWAS data, if available, rather than childhood seropositivity. Anti-*H. pylori* IgG reflects prior immunological exposure but does not necessarily indicate current or chronic infection, which may be more biologically relevant to pancreatic cancer risk. Because GWAS for more specific or mechanistically informative serologic markers (e.g., Cytotoxin-associated gene A (CagA)/Vacuolating cytotoxin A (VacA) positivity) are not yet available, our analysis necessarily relies on anti-IgG levels as the only instrumentable exposure, and this limitation may attenuate causal estimates or introduce exposure misclassification. Expanding outcome datasets by including additional international pancreatic cancer consortia would improve power to detect modest causal effects. Multivariable or two-step MR approaches could explore whether any indirect effect of *H. pylori* operates through gastric cancer or metabolic risk factors. The possibility of sample overlap can be excluded, as the exposure and outcome datasets originate from distinct populations with non-overlapping birth cohorts and recruitment ages.

Future Mendelian randomisation studies would be substantially enhanced by the availability of genome-wide association studies of adult serological phenotypes, genome-wide association studies of strain-specific virulence factors such as CagA and VacA, and the application of multi-antigen seropositivity panels capable of more accurately distinguishing chronic colonisation from transient infection. Finally, subtype-specific analyses of pancreatic cancer and functional annotation of *H. pylori*–related SNPs may reveal biologically relevant pathways that are obscured in aggregate analyses.

## 5. Conclusions

In this two-sample Mendelian randomisation study, we found no statistically significant evidence for a causal relationship between *Helicobacter pylori* infection and pancreatic cancer risk. However, across both the UK Biobank and the combined FinnGen–MVP–UK Biobank datasets, odds ratios derived from multiple analytical methods trended in a similar positive direction. This suggests that a weak causal effect cannot be entirely ruled out.

The absence of statistical significance likely reflects limitations in study power, driven by the relatively small number of pancreatic cancer cases available for analysis and the use of genetic instruments derived from SNPs that did not meet conventional genome-wide significance thresholds. Furthermore, the reliance on childhood seropositivity data as a proxy for chronic or adult infection may have reduced the biological relevance of the exposure instruments.

Taken together, these findings suggest that *H. pylori* is unlikely to be a major causal factor in pancreatic cancer development, but the possibility of modest effects remains open. Future Mendelian randomisation studies with larger sample sizes, genome-wide significant instruments for *H. pylori*, and more precise exposure measures will be important to confirm or refute this potential association.

## Data Availability

The datasets analysed during the current study are available in the IEU Open GWAS (https://gwas.mrcieu.ac.uk/datasets/ieu-b-4905/ (accessed 20 March 2025)). The outcome summary statistics can be accessed by applying at: https://storage.googleapis.com/finngen-public-data-r12/meta_analysis/mvp_ukbb/summary_stats_filtered/C3_PANCREAS_EXALLC_meta_out_filtered.tsv.gz (accessed 20 March 2025).

## Supplementary Materials

The following supporting information can be downloaded at: https://www.mdpi.com/article/10.3390/life16020284/s1, Table S1: Instrument variables of anti-*H. pylori* IgG levels on PaCa two-sample MR analysis in UKBB; Table S2: Instrument variables of anti-*H. pylori* IgG levels on PaCa two-sample MR analysis in the combined dataset (FinnGen + UKBB + MVP); Table S3: Instrument variables of anti-*H. pylori* IgG levels on PaCa two-sample MR analysis in FinnGen; Table S4: Instrument variables of anti-*H. pylori* IgG levels on PaCa two-sample MR analysis in MVP (EUR). Table S5: MR analyses results in MVP (EUR) and FinnGen; Table S6: Pancreatic cancer GWAS dataset.

## Abbreviations

The following abbreviations are used in this manuscript:

2SMR	Two-sample Mendelian randomisation
PaCa	Pancreatic cancer
*H. pylori*	*Helicobacter pylori*
IVW	Inverse-variance weighted
GWAS	Genome-wide association study
IV	Instrumental variable
MR-PRESSO	Mendelian Randomisation Pleiotropy RESidual Sum and Outlier
SNPs	Single-nucleotide polymorphisms
WM	Weighted median
WMO	Weighted mode
UKBB	UK Biobank
